# Depression, malnutrition, and health-related quality of life among Nepali older patients

**DOI:** 10.1186/s12877-018-0881-5

**Published:** 2018-08-24

**Authors:** Saruna Ghimire, Binaya Kumar Baral, Buddhi Raj Pokhrel, Asmita Pokhrel, Anushree Acharya, Dipta Amatya, Prabisha Amatya, Shiva Raj Mishra

**Affiliations:** 1 Agrata Health and Education (AHEAD)-Nepal, Kathmandu, Nepal; 2 Department of Biochemistry, Nepal Medical College and Teaching Hospital, Kathmandu, Nepal; 3 Department of Nutrition and Dietetics, College of Applied Food and Dairy Technology, Purbanchal University, Kathmandu, Nepal; 4 Nepal Development Society, Bharatpur-10, Nepal

**Keywords:** Nutritional assessment, MNA, Depression, Quality of life, Elderly, Nepal, Mediation, Moderation

## Abstract

**Background:**

Little is known about the health, nutrition, and quality of life of the aging population in Nepal. Consequently, we aimed to assess the nutritional status, depression and health-related quality of life (HRQOL) of Nepali older patients and evaluate the associated factors. Furthermore, a secondary aim was to investigate the proposed mediation-moderation models between depression, nutrition, and HRQOL.

**Methods:**

A cross-sectional survey was conducted from January–April of 2017 among 289 Nepali older patients in an outpatient clinic at Nepal Medical College in Kathmandu. Nutritional status, depression and HRQOL were assessed using a mini nutritional assessment, geriatric depression scales, and the European quality of life tool, respectively. Linear regression models were used to find the factors associated with nutritional status, depression, and HRQOL. The potential mediating and moderating role of nutritional status on the relationship between depression and HRQOL was explored; likewise, for depression on the relationship between nutritional status and HRQOL.

**Results:**

The prevalence of malnutrition and depression was 10% and 57.4% respectively; depression-malnutrition comorbidity was 7%. After adjusting for age and gender, nutritional score (β = 2.87; BCa 95%CI = 2.12, 3.62) was positively associated and depression score (β = − 1.23; BCa 95%CI = − 1.72, − 0.72) was negatively associated with HRQOL. After controlling for covariates, nutritional status mediated 41% of the total effect of depression on HRQOL, while depression mediated 6.0% of the total effect of the nutrition on HRQOL.

**Conclusions:**

A sizeable proportion of older patients had malnutrition and depression. Given that nutritional status had a significant direct (independently) and indirect (as a mediator) effect on HRQOL, we believe that nutritional screening and optimal nutrition among the older patients can make a significant contribution to the health and well-being of Nepali older patients. Nonetheless, these findings should be replicated in prospective studies before generalization.

## Background

The population of older adults, 60 years and above, in Nepal has increased from 1.5 million to 2.2 million in recent years [1, 2]. The 3.5% population growth rate of the older adults from 2001 to 2011 is higher than the population growth rate (2%) of the overall country [1–3], which hints at a slowly shifting demographic structure in Nepal concomitant with overall gains in life expectancy. Notably, this growth in life expectancy (10 years gain in the last 20 years) carries a disease burden. Malnutrition and depression are known major problems amongst senior citizens, contributing significantly to decreased health-related quality of life (HRQOL) [4, 5]. Yet, little is known about the health, nutrition, and HRQOL of Nepali older adults.

The national prevalence of malnutrition among Nepali older adults is entirely unknown, although one study conducted in rural Nepal found an estimated 24% prevalence of malnutrition among older adults [6]. The current study, conducted among urban older patients, will supplement the previous nutritional assessment in rural Nepal [6] to provide more comprehensive knowledge on this important issue. Previous studies examining the prevalence of depression among segments of Nepal’s older population found estimates ranging from 47 to 53% [7, 8]. In the absence of large nationally representative studies, small studies conducted in diverse settings, such as the current and previous studies [7, 8], can serve to provide valuable baseline information on depression status and its correlates among the older patients. Aging is one of the most important causes of decreasing HRQOL and wellbeing due to biological senescence and socio-psychological changes [9]. Although HRQOL indicators have played a major role in the development of health services globally [10], this is relatively uncommon in Nepal. Moreover, studies assessing the HRQOL of the burgeoning older population in Nepal are lacking. One previous study reported low HRQOL among older adults [11]; however, the study used a relatively homogenous study population: predominantly female visually impaired nursing home residents, reducing the generalizability of their findings.

In 2010, a comprehensive review by the Nepal Geriatric Centre [3] for the Ministry of Health and Population in Nepal highlighted the lack of studies on the health, nutritional state, and overall HRQOL of older adults in Nepal. They recommended continued research to fill these gaps in knowledge in order to effectively be able to plan programs and interventions that maximize the HRQOL of the older population in Nepal. Therefore, our primary aim was to assess the status of nutrition, depression, and HRQOL among Nepali older patients and identify factors that are associated with these outcomes.

**Fig. 1:**
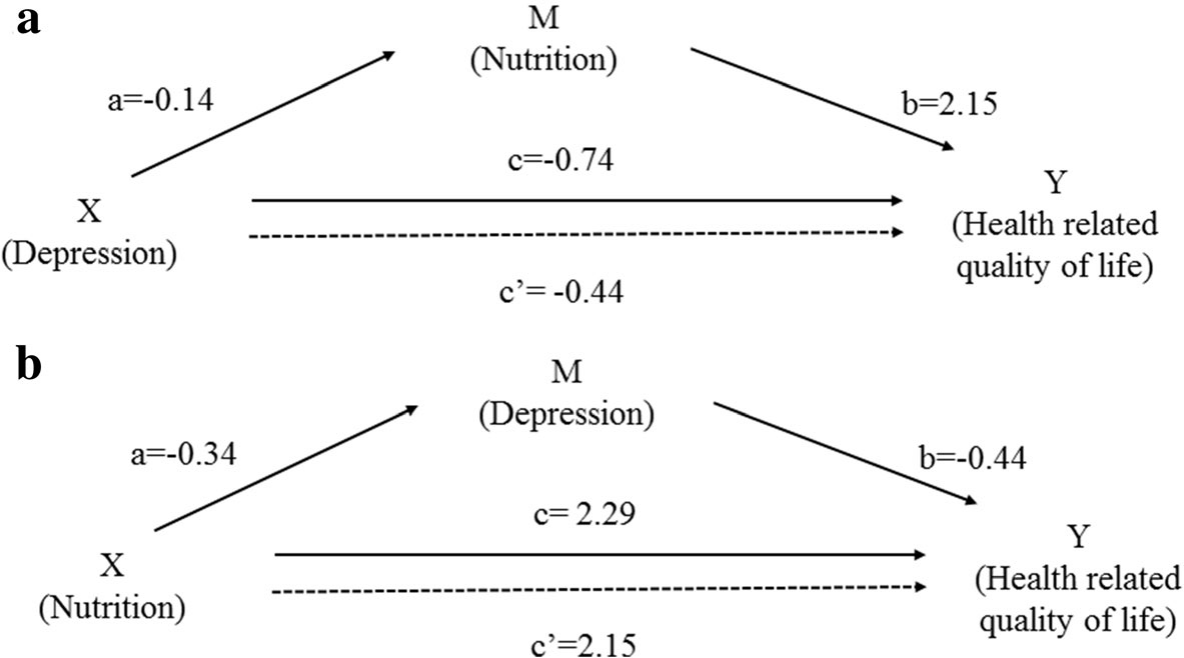
Mediation model **a** for the association between depression and health related quality of life, mediated by nutrition; **b** for the association between nutrition and health related quality of life, mediated by depression. X: independent variable; Y: outcome variable; M: mediator variable; a: association between independent variable (X) and potential mediator (M); b: association between potential mediator (M) and outcome variable (Y), controlling for independent variable (X); c: total effect of the independent variable (X) on outcome variable (Y); c’: direct effect (unmediated) of independent variable (X) on outcome variable (Y). Model is adjusted for age, sex, ethnicity, marital status, smoking, alcohol use, educational status, perception of negligence/hatred, perceived health status compared to others

**Fig. 2:**
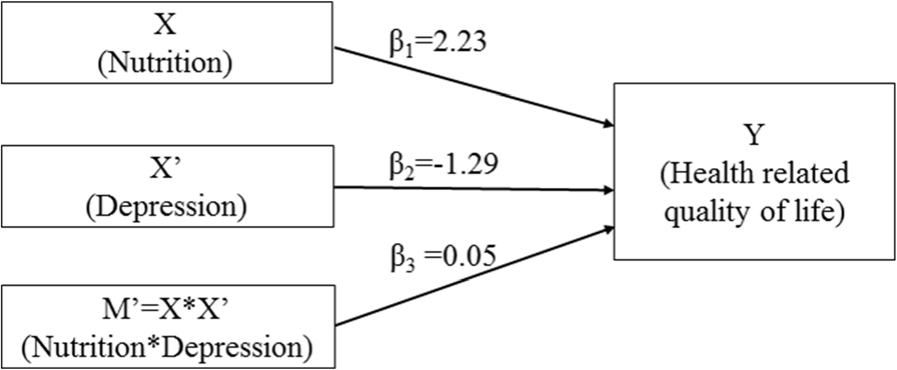
Moderation model for the moderating effect of nutrition and depression on health-related quality of life. X and X’: independent variables; M’: moderation between the independent variables nutrition and depression; Y: outcome variable; β_1_: association between nutrition (X) and health-related quality of life (Y); β_2_: association between depression (X’) and health-related quality of life (Y); β_3_: moderation effect of nutrition and depression on health-related quality of life. Unadjusted model

Our secondary aim was to evaluate the depression-nutrition-HRQOL triad in mediation-moderation models (Figs. 1 and 2). We hypothesized that both depression and malnutrition would have a significant negative impact on HRQOL among the older patients in Nepal. In addition to finding a bidirectional link between nutritional status and depression [4, 12, 13], previous studies have shown that depression and nutrition independently contribute to decreased HRQOL among older adults [4, 5]. Therefore, based on the literature, it is plausible that additional moderating or mediating effects may be present in the nutrition-depression-HRQOL triad; studies exploring such mediation-moderation effects are lacking. Exploring these pathways and determining which pathway is more plausible will enrich our understanding of the HRQOL of the older adults. More importantly, it will aide in devising effective interventions to promote HRQOL and healthy aging among the older adults.

## Methods

### Study procedure

This study, abbreviated as NepEldQOL I, supplements our previous study, NepEldQOL II [14]; together these studies provide the most comprehensive portrayal of the well-being of Nepal’s older population to date. The current study was conducted in January–April of 2017 in the outpatient department (OPD) of Nepal Medical College and Teaching Hospital (NMCTH) in Kathmandu, Nepal. According to hospital administration data, NMCTH had a total of 138,684 outpatient visits in 2016–2017; of these, 16,567 visits were among patients aged 60 years and above.

The required sample size of 289 was calculated by using StatCalc in Epi Info 7 based on a 24% prevalence of malnutrition among Nepali older adults [6], 5% alpha or Type I error and a 5% margin of error. Surveyors were graduate students in medicine and public health who were provided with a one-day extensive orientation on the study tools, sampling strategy, and data collection techniques. Each day, participants were selected by systematic random sampling from the daily “first-come first served” OPD sign-in lists, since NMCTH, like most health service facilities in Nepal, uses only a walk-in appointment system for all new or revisiting patients. The daily OPD record (updated continuously throughout the day) served as the population sample reference frame for each day of data collection, which took place during normal business hours from January to mid-April, when the required sample size was achieved. Surveyors identified patients ages 60 and above from the OPD list, and subsequently approached every third patient to screen for eligibility. Eligible participants were 60 years or older and present in the OPD one on the data collection days. Those too frail physically or mentally to respond, and/or with hearing or speech impairment were excluded. Consent to participate was requested from all eligible patients. If the approached patient was deemed ineligible or if s/he refused to participate, the next eligible patient from the OPD list was approached. Of the 297 patients approached, eight eligible patients refused to participate; the remainder granted consent.

### Data collection and variables

Individual face-to-face interviews were conducted with patients in the waiting area of the OPD. The survey tools were translated from English to Nepali by the second author and then verified by back-translation to English by another author, following the protocol for translations [15]. Any disagreement on translation was resolved by adjudication by a third author and mutual consensus between the three authors.

### Nutritional assessment

Nutritional status was assessed using the Short Form Mini Nutritional Assessment (MNA), validated previously among Nepali older adults [6]. For the measurement of BMI, the surveyors measured each participant’s height with a mechanical stadiometer (Prestige HM 007) and weight with a digital weighing scale (SECA GMBH & Co Model: 874). BMI was calculated as weight in kg/(height in m)^2^ which was categorized as per instructions in the MNA guide [16]. The cumulative MNA score ranges from 0 to 14. A score of less than 8 indicates malnourishment, a score between 8 and 11 points indicates that the subject is at risk of malnutrition and a score of 12 or higher indicates a normal nutritional status. Details on the MNA tool are provided elsewhere [16]; briefly, the MNA short form consists of six items: decline in food intake, involuntary weight loss, mobility, psychological stress, neuropsychological problems and body mass index (BMI). While the World Health Organization defines malnutrition as “deficiencies, excesses, or imbalances in a person’s intake of energy and/or nutrients” [17], we use the words “nutritional status” and “malnourished” throughout the manuscript to reflect only deficient nutritional status in order to be consistent with the MNA tool. The Cronbach’s Alpha, which is the measurement of scale reliability, for the MNA scale in the current study was 0.59.

### Depression assessment

The Geriatric Depression Scale Short Form (GDS) was used to measure depression [18]. The GDS has been described in detail elsewhere [18]; briefly, it is a 15-item instrument with responses in “Yes/No” format. Of the 15 items, 10 indicate the presence of depression when answered positively while the other five are indicative of depression when answered negatively (reverse coded for cumulation). A cumulative score of 5 or more suggests depression [19]. The validity and reliability of GDS to measure depression among community-dwelling Nepali older adults has been established by a previous study [20]. The Cronbach’s Alpha for the GDS was 0.81 in the current study.

### Health-related quality of life

HRQOL was assessed using the European quality of life tool (EQ-5D) [21]. The Nepali versions of the EQ-5D tools have been validated in a previous study [22]. The EQ-5D allows participants to classify their health status in five different dimensions (i.e., mobility, self-care, usual activities, pain/discomfort and anxiety/depression) and within a three-level response (no problems, moderate problems, and severe problems) [21]. The five dimensions of the ED-5D are then converted into a single index value, called EQ-5D index, by using the “EQ-5D-3L crosswalk index value calculator” [23], using United Kingdom (UK) weights as the reference. The EQ-5D index ranges from 0 to 1, where 0 indicates severely ill, and 1 indicates perfect health. Perfect health is represented by no problems across all five dimensions; severely ill corresponds to severe problems on all five dimensions of EQ-5D.

Additionally, EQ-5D has a vertically calibrated scale called the EuroQol visual analytic scale (EQVAS), which allows participants to rate their overall health on a scale ranging from 0 to 100, where 0 and 100 signify the worst and the best imaginable health state level, respectively. Participants rated their overall health in EQVAS at the level they felt best described their health on the study day. For this study, the Cronbach’s Alpha of the EQ-5D scale was 0.79.

### Sociodemographic variables

Sociodemographic variables were self-reported and included age, gender, ethnicity, religion, marital status, educational status, occupation, monthly family income, family structure, smoking, and alcohol use. For ethnicity, the Nepal Health Management Information System’s ‘caste/ethnic groupings’ were used [24]. Due to sparsity in certain categories, related categories were combined to form three categories: Upper caste, Janjatis, and Dalit/other minorities. Education status was categorized into three groups: illiterate; informal (no formal schooling, some literacy); and formal education (any years of formal schooling). Occupation indicated the primary occupation of the participant in the past or the current occupation if currently employed (*n* = 27). Adequate rest was defined as sleeping for more than six hours daily. Information on primary caretakers was collected through open-ended questioning and recoded into two categories: son and daughter-in-law as the first; others as the second.

### Statistical analyses

Statistical analyses were performed in IBM SPSS v22. Numerical variables are expressed as a mean and standard deviation (SD); categorical variables as frequency and percentage. Comparisons of means between the groups were made by independent t-tests or analysis of variance, while frequency distributions were evaluated by Pearson’s chi-square (χ^2^) test. The Spearman correlation coefficient (ϱ) was calculated to estimate the correlation of EQVAS with total MNA and GDS scores. The factors associated with nutritional status, depression, and HRQOL were assessed by three separate linear regression models, each adjusted for age and sex, using the total score of MNA, GDS, and EQVAS as the dependent variable, respectively. To account for non-normally distributed outcome variables and a relatively small sample size, bootstrap models with 5000 replications were used for calculating stable estimates of correlates and their bias accelerated and corrected (BCa) 95% confidence intervals.

Two different mediating models were developed for the depression-nutrition-HRQOL triad (Fig. 1): the first uses total MNA score, or nutritional status, as the mediator (Fig. 1a) while the second uses total GDS score, or depression status, as the mediator (Fig. 1b). For moderation analyses (Fig. 2), an interaction between depression and nutritional score was added to a base regression model with depression and nutritional score as independent factors; EQVAS was used as the outcome. The PROCESS macro for SPSS was used for the mediation-moderation analyses. The mediation analyses were first run without any covariates (Model 1); then adjusted for age and sex (Model 2); then further adjusted for ethnicity, marital status, smoking, alcohol use, educational status, perception of negligence/hatred, and perceived health status compared to peers (Model 3). For all statistical tests, two-tailed *p*-values< 0.05 were considered statistically significant.

## Results

### Demographic profile of the participants

Detailed characteristics of the study participants can be found in Table 1. A total of 170 males and 119 females participated. The mean age of participants was 68.5 years, with a range from 60 to 90 years old. Most were Upper caste (46%) or Janjatis (46%), Hindu (76%), married (80%), illiterate (39%), and lived in a joint family (70.6%). The primary past occupation among males was farming (39%); among females it was household chores (48%). Many participants were reluctant to reveal their monthly family income; among respondents (*n* = 167), the mean monthly family income was $205. Only a small proportion (10%) were actively involved in earnings; over half of the participants were taken care by their son and daughter-in-law. The mean (±SD) BMI, EQVAS, MNA, and GDS scores of the participants were 24.9 ± 3.5, 65.2 ± 16.8, 10.9 ± 2.5, and 5.9 ± 3.8, respectively (Table 1).

**Table 1:** The subjects’ characteristics according to sex

	Total (*n* = 289)	Male (*n* = 170)	Female (*n* = 119)	*p* value
	n (%)	n (%)	n (%)	
Age, (mean ± SD)	68.5 ± 6.5	69.5 ± 6.6	67.1 ± 6.1	**0.002** ^a^
Gender				
Male	170 (58.8)			
Female	119 (41.2)			
Ethnicity				
Upper caste	134 (46.4)	84 (49.4)	50 (42.0)	0.429
Janjatis	134 (46.4)	75 (44.1)	59 (49.6)	
Dalit and minorities	21 (7.3)	11 (6.5)	10 (8.4)	
Religion				0.656
Hindu	220 (76.1)	131 (77.1)	89 (74.8)	
Non-Hindu	69 (23.9)	39 (22.9)	30 (25.2)	
Marital status				0.138
Married	231 (79.9)	141 (82.9)	90 (75.6)	
Separated/Widow/Single	58 (20.1)	29 (17.1)	29 (24.4)	
Educational status				**< 0.001**
Illiterate	112 (38.8)	46 (27.1)	66 (55.5)	
Informal	97 (33.6)	66 (38.8)	31 (26.1)	
Formal	80 (27.7)	58 (34.1)	22 (18.5)	
Past Occupation				**< 0.001**
Agriculture	114 (39.4)	73 (42.9)	41 (34.5)	
Homemaker	57 (19.7)	–	57 (47.9)	
Business/job	93 (32.2)	75 (44.1)	18 (15.1)	
Others	25 (8.7)	22 (12.9)	3 (2.5)	
Monthly family income (n = 167), $, (mean ± SD)	205.3 ± 90.1	206.8 ± 88.8	203.1 ± 92.7	0.799^a^
Family Structure				**0.013**
Nuclear	47 (16.3)	21 (12.4)	26 (21.8)	
Joint	204 (70.6)	120 (70.6)	84 (70.6)	
Extended	38 (13.1)	29 (17.1)	9 (7.6)	
Smoking				0.102
Yes	155 (53.6)	98 (57.6)	57 (47.9)	
No	134 (46.4)	72 (42.4)	62 (52.1)	
Alcohol use				0.397
Yes	98 (33.9)	61 (35.9)	37 (31.1)	
No	191 (66.1)	109 (64.1)	82 (68.9)	
Self-rated health status				0.380
Better	69 (23.9)	40 (23.5)	29 (24.4)	
Similar	139 (48.1)	87 (51.2)	52 (43.7)	
Worse	81 (28.0)	43 (25.3)	38 (31.9)	
Adequate rest				**0.042**
Yes	81 (28.0)	40 (23.5)	41 (34.5)	
No	208 (72.0)	130 (76.5)	78 (65.5)	
Working currently				**0.012**
Yes	27 (9.3)	22 (12.9)	5 (4.2)	
No	262 (90.7)	148 (87.1)	114 (95.8)	
Caretaker				**0.004**
Son and daughter in law	153 (52.9)	78 (45.9)	75 (63.0)	
Others	136 (47.1)	92 (54.1)	44 (37.0)	
Ignored/hated for being old				**0.005**
Yes	45 (15.6)	18 (10.6)	27 (22.7)	
No	244 (84.4)	152 (89.4)	92 (77.3)	
BMI, kg/m^2^, (mean ± SD)	24.9 ± 3.5	24.8 ± 3.5	24.9 ± 3.7	0.837^a^
MNA score, (mean ± SD)	10.9 ± 2.5	11.2 ± 2.5	10.7 ± 2.5	0.099^a^
GDS Score, (mean ± SD)	5.9 ± 3.8	5.9 ± 3.9	5.8 ± 3.7	0.827^a^
EQVAS, (mean ± SD)	65.2 ± 16.8	66.4 ± 17.3	63.6 ± 16.1	0.179^a^

^a^*p*-value from independent t-test test; all others are chi-square. 1$ = 100 Nepalese rupees

Abbreviation: *SD* standard deviation, *BMI* body mass index, *MNA* mini nutritional assessment short form cumulative score, *GDS* Geriatric depression scale short form cumulative score, *EQVAS* European quality of life visual analytical scale

### Prevalence and correlates of nutritional status

Appendix Table 5 provides the detailed characteristics of participants based on nutritional status categories as defined by MNA. The mean MNA score was 11.0 and ranged from 2 to 14. Only about half of the participants had adequate nutritional status; 10% were malnourished, and 38% were at risk of malnutrition. Comorbidity between depression and malnutrition was prevalent among 6.9% of the participants (Appendix Table 5). In the regression analysis adjusted for age and sex (Table 2), age (β = − 0.07; BCa 95%CI = − 0.12, − 0.01), male gender (β = − 0.65; BCa 95%CI = − 1.25, − 0.07), depression score (β = − 0.18; BCa 95%CI = − 0.26, − 0.10), perception of worsened health (β = − 1.04; BCa 95%CI = − 1.73, − 0.33) and perception of ignorance/hatred due to old age (β = − 1.92; BCa 95%CI = − 2.73, − 1.09) were inversely associated with the nutritional score from the MNA. Likewise, having formal education (β = 0.81; BCa 95%CI = 0.18, 1.47) as well as higher quality of life scores on both the EQ-5D index (β = 2.98; BCa 95%CI = 1.51, 4.30) and the EQVAS (β = 0.06; BCa 95%CI = 0.05, 0.08) were associated with a higher nutritional score on the MNA.

**Table 2:** Factors associated with nutritional status, depression, and health-related quality of life

	MNA		GDS		EQVAS	
	β	BCa 95% CI	β	BCa 95% CI	β	BCa 95% CI
Age	**−0.07**	**− 0.12, − 0.01**	**0.10**	**0.03, 0.17**	− 0.20	− 0.54, 0.11
Gender (Reference = Male)	**− 0.65**	**−1.25, − 0.07**	0.14	− 0.74, 1.03	−3.18	−7.35, 0.82
Ethnicity (Reference = Upper caste)						
Janjatis	− 0.02	− 0.60, 0.56	0.39	− 0.46, 1.24	0.03	− 3.82, 3.87
Dalit and minorities	− 0.78	− 1.97, 0.41	1.55	− 0.10, 3.19	−2.37	− 10.67, 5.37
Marital status (Reference = Separated/Widow/single)	0.48	− 0.24, 1.18	− 0.48	− 1.61, 0.62	**6.57**	**1.75, 11.37**
Education (Reference = Illiterate)						
Informal	0.50	−0.09, 1.07	**1.06**	**0.16, 1.96**	−0.37	−4.48, 3.61
Formal	**0.81**	**0.18, 1.47**	**−1.43**	**−2.43, −0.40**	**5.54**	**0.92, 9.99**
Smoking (Reference = No)	0.10	−0.47, 0.65	−0.08	− 0.97, 0.76	−2.29	−6.20, 1.49
Alcohol use (Reference = No)	−0.06	− 0.63, 0.48	−0.04	− 0.95, 0.89	0.39	−3.64, 4.27
Adequate rest everyday (Reference = No)	0.17	−0.56, 0.88	−0.28	−1.27, 0.71	0.01	−4.92, 4.89
Family type (Reference = Joint)						
Nuclear	−0.31	−1.16, 0.51	0.52	−0.68, 1.73	−4.89	−10.24, 0.45
Extended	0.19	−0.63, 0.94	0.94	−0.30, 2.20	−3.01	−8.97, 2.50
Currently working (Reference = No)	0.75	−0.35, 1.77	−0.90	−2.32, 0.62	**11.60**	**4.57, 18.07**
Care taker (Reference = Son)	0.36	−0.25, 0.96	−0.38	−1.27, 0.51	0.18	−3.68, 4.08
Self-perceived health status (Reference = Similar)						
Better	0.21	−0.48, 0.88	−0.37	−1.39, 0.70	**11.06**	**7.34, 15.03**
Worse	**−1.04**	**−1.73, −0.33**	**1.68**	**0.74, 2.60**	**−18.20**	**−22.34, − 13.94**
Ignored/hated for being old (Reference = No)	**− 1.92**	**−2.73, − 1.09**	**1.58**	**0.32, 2.82**	**−8.63**	**−13.61, −3.80**
MNA	–		**−0.41**	**−0.59, − 0.23**	**2.87**	**2.12, 3.62**
GDS	**−0.18**	**−0.26, − 0.10**	–		**−1.23**	**− 1.72, − 0.72**
EQ-5D Index	**2.98**	**1.51, 4.30**	**−2.49**	**−4.61, − 0.48**	**33.86**	**25.41, 42.61**
EQVAS	**0.06**	**0.05, 0.08**	**−0.06**	**−0.09, − 0.03**	–	

β: Unstandardized coefficient; BCa: Bias-corrected and accelerated

Adjusted for age and sex

Number of bootstrap samples for bias-corrected bootstrap confidence intervals: 5000

Statistically significant associations are highlighted in bold

Abbreviation: *MNA* mini nutritional assessment short form cumulative score, *GDS* Geriatric depression scale short form cumulative score, *EQVAS* European quality of life visual analytical scale

### Prevalence and correlates of depression

Appendix Table 6 provides the detailed characteristics of participants based on depression status as defined by GDS. More than half (57%) of the participants met the criteria of depression (GDS score ≥ 5). In the regression analysis adjusted for age and sex (Table 2), higher age (β = 0.10; BCa 95%CI = 0.03, 0.17), perception of worsened health (β = 1.68; BCa 95%CI = 0.74, 2.60) and perception of ignorance/hatred due to old age (β = 1.58; BCa 95%CI = 0.32, 2.82) were associated with a higher depression score. Compared to illiterate individuals, those having an informal education (β = 1.06; BCa 95%CI = 0.16, 1.96) scored higher on the depression scale whereas those having a formal education (β = − 1.43; BCa 95%CI = − 2.43, − 0.40) scored lower. A higher depression score on the GDS was associated with a lower score on the nutrition scale (β = − 0.41; BCa 95%CI = − 0.59, − 0.23) and lower quality of life scores: EQ-5D index (β = − 2.49; BCa 95%CI = − 4.61, − 0.48) and EQVAS (β = − 0.06; BCa 95%CI = − 0.09, − 0.03).

### Health-related quality of life and its correlates

The responses of participants in the five dimensions of EQ-5D are provided in Appendix Table 7. The mean EQVAS score and the EQ-5D index were 65.2 and 0.8 respectively; scores were significantly lower among participants meeting the criteria for malnutrition or depression. Thirty-six different health statuses were represented in the EQ-5D (Appendix Table 7).

EQVAS scores were positively correlated with MNA scores (ϱ =0.44, *p* < 0.001) and negatively correlated with GDS scores (ϱ = − 0.28, *p* < 0.001). In the regression analysis adjusted for age and sex using EQVAS as the outcome (Table 2), a positive association was observed between the EQVAS QOL score and being married (β = 6.57; BCa 95%CI = 1.75, 11.37), having a formal education (β = 5.54; BCa 95%CI = 0.92, 9.99), working currently (β = 11.60; BCa 95%CI = 4.57, 18.07), better perceived health status (β = 11.06; BCa 95%CI = 7.34, 15.03), and higher MNA score (β = 2.87; BCa 95%CI = 2.12, 3.62). Likewise, an inverse association was observed between the EQVAS QOL score and perception of worsen health status (β = − 18.20; BCa 95%CI = − 22.34, − 13.94), perception of being ignored/hated for old age (β = − 8.63; BCa 95%CI = − 13.61, − 3.80), and the depression score (β = − 1.23; BCa 95%CI = − 1.72, − 0.72).

### Mediation-moderation analysis

#### Nutrition as a mediator of the depression – health-related quality of life association

Table 3 and Fig. 1a present the findings from mediation analysis, exploring the depression-nutrition-HRQOL pathway using the MNA score as the mediator. In the unadjusted analysis, the ratio of depression’s indirect effect to the total effect through nutrition was 40%. In the final adjusted model (Model 3), nutritional score mediated 41% of the total effect of depression on HRQOL (Table 3).

**Table 3:** Mediation analysis for the association between depression and health-related quality of life, mediated by nutrition

	Model 1		Model 2		Model 3	
	β	BCa 95% CI	β	BCa 95% CI	β	BCa 95% CI
Total effect, c	−1.25 (0.25)	−1.75, − 0.75	−1.23 (0.26)	− 1.74, − 0.72	−0.74 (0.24)	−1.20, − 0.28
Direct effect, c’	−0.74 (0.25)	−1.23, − 0.26	−0.76 (0.25)	−1.25, − 0.28	−0.44 (0.23)	− 0.88, 0.01
Indirect effect, ab	−0.50 (0.14)	− 0.83, − 0.27	−0.47 (0.14)	− 0.77, − 0.24	−0.31 (0.11)	− 0.56, − 0.12
Ratio of indirect to total effect mediated	0.40		0.38		0.41	
Ratio of indirect to direct effect	0.68		0.61		0.70	

Model 1: Unadjusted mediational model

Model 2: Adjusted for age, and sex

Model 3: Adjusted for age, sex, ethnicity, marital status, smoking, alcohol use, educational status, perception of negligence/hatred, perceived health status compared to others

Number of bootstrap samples for bias-corrected bootstrap confidence intervals: 5000

β: Unstandardized coefficient; BCa: Bias-corrected and accelerated

#### Depression as a mediator of the nutrition – health-related quality of life association

Table 4 and Fig. 1b present the findings from the mediation analysis exploring the nutrition- depression-HRQOL pathway with depression as the mediator. In the unadjusted analysis, depression score mediated 11% of the total effect of nutritional score on HRQOL; this dropped to 6.0% in the final adjusted model (Model 3).

**Table 4:** Mediation analysis for the association between nutrition and health-related quality of life, mediated by depression

	Model 1		Model 2		Model 3	
	β	BCa 95% CI	β	BCa 95% CI	β	BCa 95% CI
Total effect, c	2.90 (0.35)	2.20, 3.60	2.87 (0.36)	2.16, 3.58	2.29 (0.34)	1.62, 2.97
Direct effect, c’	2.58 (0.37)	1.86, 3.30	2.56 (0.37)	1.83, 3.29	2.14 (0.35)	1.45, 2.84
Indirect effect, ab	0.32 (0.13)	0.12, 0.62	0.31 (0.12)	0.12, 0.60	0.15 (0.09)	0.02, 0.38
Ratio of indirect to total effect mediated, (ab/c)	0.11		0.11		0.06	
Ratio of indirect to direct effect, (ab/c’)	0.13		0.12		0.07	

Model 1: unadjusted mediational model

Model 2: Adjusted for age, and sex

Model 3: Adjusted for age, sex, ethnicity, marital status, smoking, alcohol use, educational status, perception of negligence/hatred, perceived health status compared to peers

Number of bootstrap samples for bias-corrected bootstrap confidence intervals: 5000

β: Unstandardized coefficient; BCa: Bias-corrected and accelerated

##### Moderation analyses

In an unadjusted moderation analysis (Fig. 2), the interaction between MNA and GDS (β = 0.05; BCa 95%CI = − 0.16, 0.26) was not significantly associated with HRQOL.

## Discussion

In this study, we assessed the nutrition status, depression status, and HRQOL of Nepalese community-dwelling older patients in urban Kathmandu. We also explored the differential effects of nutrition, depression, and HRQOL by mediation and moderation analyses. A sizeable proportion of our study population had prevalent malnutrition and depression.

In the current study, nutritional and depression were inversely related to each other; many malnourished individuals were depressed and vice versa. Although studies quantifying the relationship between nutrition and depression in older adults in Nepal are lacking, studies from Iran [4, 12], Norway [25], and Brazil [26] support our findings. The link between poor nutrition and depression is biologically plausible [27, 28]: multiple pathways such as inflammation, oxidative and nitrosative stress, as well as a decrease in antioxidant levels [27, 28] support the underlying role of several nutrients in explaining the mechanism of depression.

Our mediation analyses suggested that in the depression-nutrition-HRQOL triad, both nutrition and depression partially mediate each others association with HRQOL; however, nutritional status mediated a greater proportion of the total effect on HRQOL in comparison to depression. Not only did poor nutritional status have a significant direct effect on HRQOL, but it also partially explained the relationship between depression and HRQOL. Patients with depression are more likely to exhibit loss of appetite, decreased food intake, meal skipping, and disordered eating; which can lead to poor nutritional outcomes and vice-versa [29]. Likewise, in previous studies, nutritional risk was found to be a significant factor associated with HRQOL [4, 30]; nutritional wellbeing can influence HRQOL by affecting functional ability, muscle mass, and formation and transportation of proteins and hormones [31]. In previous studies, depressive symptoms and impaired nutritional status were independently associated with lower HRQOL scores among the older adults [4, 5]. Our previous study, NepEldQOL II [14], also suggested a potential mediating role of depression in the relationship between nutrition and life-satisfaction. The current study provided preliminary evidence to support the role of nutritional status in maintaining optimal HRQOL among the older patients. Public health interventions for optimizing HRQOL should consider screening for depression and nutritional status simultaneously. Prospective studies, including those that consider healthy adults at baseline, will be needed to confirm these preliminary findings.

A sizeable proportion of study participants had prevalent malnutrition and depression; findings were not unexpected. Older adults are more vulnerable to malnutrition due to age-associated changes in metabolism and/or physiological function which may cause anorexia, loss of appetite, deficits in taste and shifts in dietary choices and eating habits [32]. A previous study from rural Nepal found higher prevalence, (24% compared to our 10%) of malnutrition than this current study [6]. We had expected that the older populations in urban Nepal would have better nutritional status because households in rural areas are more likely to be food deficient [33] with higher overall poverty rates [34]. Additionally, older age, female gender, low literacy and lower family income were also associated with poor nutritional status, findings consistent with a previous study [6]. Moreover, the prevalence of depression found in our study, over 50%, is consistent with a previous hospital-based study from Kathmandu, Nepal where depression, as defined by GDS, was found among 53.2% of the older patients [8].

The mean EQVAS score and the EQ-5D index were 65.2 and 0.8 respectively. Scores were significantly lower among participants meeting the criteria for malnutrition or depression. These findings were expected among older adults given that age is the strongest predictor of HRQOL [4, 5]. Aging is characterized by a gradual and lifelong accumulation of molecular and cellular damage that subsequently leads to a decrease in physiological functions, increased vulnerability to diseases, and a general decline in the capacity of the individual [35, 36]. Furthermore, the impact of depression and poor nutrition may aggravate HRQOL among the older adults who are already susceptible to poor QOL due to their senescence.

### Strengths, limitations, and future research directions

We present a pioneer study that quantifies the HRQOL among urban Nepali older patients, filling gaps and advancing knowledge about the prevalence of and factors contributing to depression and malnutrition among Nepali older patients. Moreover, we explore the relationship between depression, nutritional status, and HRQOL, three important aspects of aging, in the Nepalese context. To our knowledge, the mediation and moderation effects of depression and nutrition with the outcome of HRQOL among the older adults have not been previously explored in any context. HRQOL, looking at health from an individual’s perspective, is truly multi-faceted as seen in this study. Simple measures to detect and treat depression among the older adults to improve their overall HRQOL should also examine nutritional wellbeing. Further prospective studies are needed to identify the direction of the relationship between depression and nutrition.

Nonetheless, this study is subject to some limitations, including a relatively small sample size. Due to our cross-sectional study design, no inferences can be made regarding the causal relationships between nutritional status, depression, and HRQOL. Future studies should determine if nutritional risk is associated with QOL over time among older adults. This study recruited participants from outpatient clinics in an urban setting; the nutritional status, depression, and HRQOL of the general population in an urban area and/or those in a rural setting may be different, thus limiting the generalizability of our findings. Exclusion of older patients who were too frail physically or mentally to respond may have resulted in a selection bias that underestimated or biased our findings towards the null. In the current study, the internal consistency of GDS and EQ-5D was high but that of the MNA scale was relatively low (Cronbach’s α = 0.59); omission of any MNA component score did not substantially increase the alpha value (data not shown). Given that the MNA has already been validated in various settings [37] as well as among Nepali older adults [6], we nonetheless believe it to be a valid tool to assess nutritional status among the older adults. The use of UK’s general population weights as reference values in the calculation of the EQ-5D index is not ideal; however, no such reference weights exist for Nepal and the same technique was used in the original study validating the Nepali version of EQ-5D [22] as well as another study from Nepal [38]. We defined adequate rest as sleeping for more than six hours daily; however, the National Sleep Foundation recommends 7–8 h of sleep for older adults [39]; thus we may have overestimated the prevalence of adequate rest. Moreover, sleep hours were self-reported. Increasing age is associated with multi-morbidity that may limit functional capacity and reduce the HRQOL [40]; presence of comorbidities were not assessed in this study. Future studies should look at the possible mediating and bi-directional relationship of multiple morbidities, nutritional status and HRQOL. Lastly, the possibility of residual confounding due to unmeasured covariates cannot be ruled out.

## Conclusions

Both malnutrition and depression were associated with HRQOL among our study population. Given that nutritional status had a significant direct and indirect (as a mediator) effect on HRQOL, we believe that nutritional screening and optimal nutrition among older patients can make a significant contribution to the overall HRQOL for older patients in Nepal. The depression management protocol should account for nutritional wellbeing as well as overall HRQOL in this population. Although we are cautious to make any causal interpretation of the findings, our study lends support to the role of optimal nutritional status and mental health in maintaining the overall health and well-being of older patients in Nepal.

## Appendix

**Table 5:** Participant’s characteristics by nutritional status

	Malnourished, n (%)	At risk of malnutrition, n (%)	Normal nutritional status, n (%)	*p*-value
Total (Prevalence)	**30 (10.4)**	**109 (37.7)**	**150 (51.9)**	
95% CI for prevalence	6.9–13.9	32.3–43.4	46.2–57.6	
Age, years, (mean ± SD)	69.6 ± 7.4	69.6 ± 7.1	67.5 ± 5.6	**0.021** ^a^
Gender				**0.028**
Male	17 (10.0)	54 (31.8)	99 (58.2)	
Female	13 (10.9)	55 (46.2)	51 (42.9)	
Ethnicity				0.157
Upper caste	13 (9.7)	43 (32.1)	78 (58.2)	
Janjatis	13 (9.7)	56 (41.8)	65 (48.5)	
Dalit and minorities	4 (19.0)	10 (47.6)	7 (33.3)	
Religion				0.270
Hindu	22 (10.0)	78 (35.5)	120 (54.5)	
Non-Hindu	8 (11.6)	31 (44.9)	30 (43.5)	
Marital status				**0.003**
Married	26 (11.3)	76 (32.9)	129 (55.8)	
Separated/Widow/Single	4 (6.9)	33 (56.9)	21 (36.2)	
Educational status				**< 0.001**
Illiterate	19 (17.0)	55 (49.1)	38 (33.9)	
Informal	8 (8.2)	33 (34.0)	56 (57.7)	
Formal	3 (3.8)	21 (26.2)	56 (70.0)	
Past Occupation				**0.008**
Agriculture	15 (13.2)	56 (49.1)	43 (37.7)	
Homemaker	4 (7.0)	21 (36.8)	32 (56.1)	
Business/job	8 (8.6)	24 (25.8)	61 (65.6)	
Others	3 (12.0)	8 (32.0)	14 (56.0)	
Monthly family income (n = 167), $, (mean ± SD)	164.4 ± 81.3	185.8 ± 92.0	222.6 ± 87.0	**0.007** ^a^
Family Structure				0.659
Nuclear	7 (14.9)	18 (38.3)	22 (46.8)	
Joint	18 (8.8)	79 (38.7)	107 (52.5)	
Extended	5 (13.2)	12 (31.6)	21 (55.3)	
Smoking				0.606
Yes	14 (9.0)	57 (36.8)	84 (54.2)	
No	16 (11.9)	52 (38.8)	66 (49.3)	
Drinker				0.432
Yes	7 (7.1)	38 (38.8)	53 (54.1)	
No	23 (12.0)	71 (37.2)	97 (50.8)	
Self-rated health status				**0.014**
Better	7 (10.1)	28 (40.6)	34 (49.3)	
Similar	8 (5.8)	48 (34.5)	83 (59.7)	
Worse	15 (18.5)	33 (40.7)	33 (40.7)	
Adequate rest				0.727
Yes	9 (11.1)	33 (40.7)	39 (48.1)	
No	21 (10.1)	76 (36.5)	111 (53.4)	
Working currently				0.047
Yes	1 (3.7)	6 (22.2)	20 (74.1)	
No	29 (11.1)	103 (39.3)	130 (49.6)	
Care taker				0.168
Son and daughter in law	20 (13.1)	60 (39.2)	73 (47.7)	
Others	10 (7.4)	49 (36.0)	77 (56.6)	
Ignored/hated for being old				**< 0.001**
Yes	13 (28.9)	20 (44.4)	12 (26.7)	
No	17 (7.0)	89 (36.5)	138 (56.6)	
Depression				**< 0.001**
Yes	20 (12.0)	78 (47.0)	68 (41.0)	
No	10 (8.1)	31 (25.2)	82 (66.7)	
BMI, kg/m^2^, (mean ± SD)	22.1 ± 3.1	24.2 ± 3.8	25.9 ± 3.0	**< 0.001** ^a^
MNA, (mean ± SD)	5.7 ± 1.2	9.7 ± 1.2	12.9 ± 0.8	**< 0.001** ^a^
GDS Score, (mean ± SD)	7.8 ± 4.0	6.8 ± 3.6	4.8 ± 3.5	**< 0.001** ^a^
EQVAS, (mean ± SD)	49.7 ± 16.3	62.5 ± 16.6	70.4 ± 14.7	**< 0.001** ^a^

Statistically significant *p*-value are highlighted in bold

^a^*p*-value from analysis of variance; all others are from Pearson’s chi-square test

Abbreviation: *SD* standard deviation, *BMI* body mass index, *MNASF* mini nutritional assessment short form cumulative score, *GDS* Geriatric Depression scale short form cumulative score, *EQVAS* European quality of life visual analytical scale

**Table 6:** Participant’s characteristics by depression status

	Depression, n (%)	No Depression, n (%)	*p* value
Total (Prevalence)	166 (57.4)	123 (42.6)	
95% CI for prevalence	51.7–63.0	37.0–48.3	
Age, years, (mean ± SD)	69.3 ± 6.6	67.5 ± 6.3	**0.020** ^a^
Gender			0.876
Male	97 (57.1)	73 (42.9)	
Female	69 (58.0)	50 (42.0)	
Ethnicity			**0.045**
Upper caste	67 (50.0)	67 (50.0)	
Janjatis	84 (62.7)	50 (37.3)	
Dalit and minorities	15 (71.4)	6 (28.6)	
Religion			0.134
Hindu	121 (55.0)	99 (45.0)	
Non-Hindu	45 (65.2)	24 (34.8)	
Marital status			0.091
Married	39 (67.2)	19 (32.8)	
Separated/Widow/Single	127 (55.0)	104 (45.0)	
Educational status			**0.007**
Illiterate	66 (58.9)	46 (41.1)	
Informal	65 (67.0)	32 (33.0)	
Formal	35 (43.8)	45 (56.3)	
Past Occupation			0.202
Agriculture	72 (63.2)	42 (36.8)	
Homemaker	35 (61.4)	22 (38.6)	
Business/job	47 (50.5)	46 (49.5)	
Others	12 (48.0)	13 (52.0)	
Monthly family income (n = 167), $, (mean ± SD)	185.8 ± 72.2	228.7 ± 103.4	**0.003** ^a^
Family Structure			0.268
Nuclear	30 (63.8)	17 (36.2)	
Joint	111 (54.4)	93 (45.6)	
Extended	25 (65.8)	13 (34.2)	
Smoking			0.639
No	75 (56.0)	59 (44.0)	
Yes	91 (58.7)	64 (41.3)	
Drinker			0.942
No	110 (57.6)	81 (42.4)	
Yes	56 (57.1)	42 (42.9)	
Self-rated health status			**0.025**
Better	33 (47.8)	36 (52.2)	
Similar	77 (55.4)	62 (44.6)	
Worse	56 (69.1)	25 (30.9)	
Adequate rest			0.900
Yes	47 (58.0)	34 (42.0)	
No	119 (57.2)	89 (42.8)	
Working currently			**0.024**
Yes	10 (37.0)	17 (63.0)	
No	156 (59.5)	106 (40.5)	
Care taker			0.223
Son and daughter in law	93 (60.8)	60 (39.2)	
Others	73 (53.7)	63 (46.3)	
Ignored/hated for being old			**0.019**
Yes	33 (73.3)	12 (26.7)	
No	133 (54.5)	111 (45.5)	
Nutritional status			**< 0.001**
Malnourished	20 (66.7)	10 (33.3)	
At risk of malnutrition	78 (71.6)	31 (28.4)	
Normal nutritional status	68 (45.3)	82 (54.7)	
BMI, kg/m^2^, (mean ± SD)	24.9 ± 3.5	24.9 ± 3.6	0.957^a^
QOL EQVAS, (mean ± SD)	61.8 ± 16.4	69.9 ± 16.4	**< 0.001** ^a^
MNASF, (mean ± SD)	10.5 ± 2.6	11.5 ± 2.3	**0.001** ^a^
GDS Score, (mean ± SD)	8.5 ± 2.6	2.3 ± 1.3	**< 0.001** ^a^

Statistically significant *p*-value are highlighted in bold

^a^*p*-value from independent t-test; all others are from Pearson’s chi-square test

Abbreviation: *SD* standard deviation, *BMI* body mass index, *MNASF* mini nutritional assessment short form cumulative score, *GDS* Geriatric Depression scale short form cumulative score, *EQVAS* European quality of life visual analytical scale

**Table 7:** Participants health related quality of life by nutritional and depression status

	Total	Malnourished	At risk of malnutrition	Normal nutritional status	*p*-value	Depression	No Depression	*p*-value
	n (%)	n (%)	n (%)	n (%)		n (%)	n (%)	
Mobility					**0.001**			**0.003**
No Problem	214 (74.0)	16 (53.3)	74 (67.9)	124 (82.7)		112 (67.5)	102 (82.9)	
Some Problem	75 (26.0)	14 (46.7)	35 (32.1)	26 (17.3)		54 (32.5)	21 (17.1)	
Self-Care					**< 0.001**			**0.002**
No Problem	238 (82.4)	18 (60.0)	81 (74.3)	139 (92.7)		127 (76.5)	111 (90.2)	
Some Problem	51 (17.6)	12 (40.0)	28 (25.7)	11 (7.3)		39 (23.5)	12 (9.8)	
Usual Activities					**< 0.001**			0.059
No Problem	217 (75.1)	14 (46.7)	73 (67.0)	130 (86.7)		116 (69.9)	101 (82.1)	
Some Problem	56 (19.4)	11 (36.7)	28 (25.7)	17 (11.3)		39 (23.5)	17 (13.8)	
Unable	16 (5.5)	5 (16.7)	8 (7.3)	3 (2.0)		11 (6.6)	5 (4.1)	
Pain/Discomfort					**< 0.001**			0.110
No pain	163 (56.4)	13 (43.3)	51 (46.8)	99 (66.0)		85 (51.2)	78 (63.4)	
moderate pain	111 (38.4)	10 (33.3)	56 (51.4)	45 (30.0)		72 (43.4)	39 (31.7)	
extreme pain	15 (5.2)	7 (23.3)	2 (1.8)	6 (4.0)		9 (5.4)	6 (4.9)	
Anxiety					**< 0.001**			0.160
None	172 (59.5)	12 (40.0)	55 (50.5)	105 (70.0)		93 (56.0)	79 (64.2)	
Moderate	117 (40.5)	18 (60.0)	54 (49.5)	45 (30.0)		73 (44.0)	44 (35.8)	
EQVAS, (mean ± SD)	65.2 ± 16.8	49.7 ± 16.3	62.5 ± 16.6	70.4 ± 14.7	**< 0.001** ^a^	61.8 ± 16.4	69.9 ± 16.4	**< 0.001** ^b^
EQ-5D Index, (mean ± SD)	0.8 ± 0.2	0.6 ± 0.4	0.7 ± 0.2	0.8 ± 0.2	**< 0.001** ^a^	0.7 ± 0.3	0.8 ± 0.2	**0.019** ^b^
No of health status in EQ5D	36	16	26	22		30	20	–
Complete health status (11111)	88 (30.4)	7 (23.3)	23 (21.1)	58 (38.7)		44 (26.5)	44 (35.8)	–

Statistically significant *p*-value are highlighted in bold

^a^*p*-value from analysis of variance; ^b^*p*-value from independent t-test; all others are from Pearson’s chi-square test

Abbreviation: *SD* standard deviation, *EQVAS* European quality of life visual analytical scale, *EQ-5D* European Quality of Life five dimension

## Abbreviations

BCa: Bias Accelerated and Corrected
BMI: Body Mass Index
CI: Confidence Intervals
EQ-5D: European Quality of Life Tool
EQVAS: European Quality of Life Visual Analytic Scale
GDS: Short Form of Geriatric Depression Scale
HRQOL: Health-Related Quality of Life
MNA: Short Form of Mini Nutritional Assessment
NMCTH: Nepal Medical College and Teaching Hospital
OPD: Outpatient Department
QOL: Quality of Life
SD: Standard Deviation
